# The role of the North Atlantic Ocean on the increase in East Asia’s spring extreme hot day occurrences across the early 2000s

**DOI:** 10.1038/s41598-024-59812-y

**Published:** 2024-04-30

**Authors:** Yong-Han Lee, Sang-Wook Yeh, Jeong-Hun Kim, Maeng-Ki Kim

**Affiliations:** 1 Department of Marine Science and Convergent Engineering, Hanyang University, Ansan, 15588 South Korea; 2 Department of Atmospheric Sciences, Kongju National University, Gongju, 32588 South Korea; 3 Earth Environment Research Center, Kongju National University, Gongju, 32588 South Korea; 4 Particle Pollution Research and Management Center, Kongju National University, Gongju, 32588 South Korea

**Keywords:** East Asia’s extreme hot day, North Atlantic Tripole-like, Linear baroclinic model, Atmospheric teleconnection, Climate sciences, Atmospheric science, Ocean sciences

## Abstract

The occurrence frequency of East Asia’s extreme hot day in boreal spring has increased since 1979. Using observational data and a Linear baroclinic model experiment, our study suggests that the occurrence of hot day is mainly due to anomalous high pressure over East Asia associated with a horizontal stationary wave train originating from a positive phase of the North Atlantic Tripole (NAT) sea surface temperature (SST) in spring. The effect of a positive phase of the NAT SST is evident in the 2000s, apparently associated with the linear trend of the North Atlantic SST like a positive phase of the NAT SST. Before 2000s, in contrast, SST forcing in the Indian Ocean and eastern tropical Pacific, which is associated with a negative phase of the NAT SST, may contribute to induce the East Asian hot days through atmospheric teleconnections. This implies that the relationship between a positive phase of the NAT SST and the occurrence of hot days in East Asia has been changed during the 2000s.

## Introduction

The properties of daytime heatwaves and tropical nights including their intensity, frequency, and duration have changed in the recent past, which is mainly due to the increase of greenhouse gas concentrations^1–6^. This extreme hot day (hot day, hereafter) can cause damage to human life as well as socio-economic aspects^7,8^. The occurrence of hot day is not limited to the regional scale, but it is observed in most regions of the Northern Hemisphere including Europe, Russia, and East Asia^9–14^.

It has been reported that the occurrence of hot day in East Asia including China, Japan, and Korea, which has a high population of more than 1.6 billion^15^, has significantly increased over the past decades^16–20^. Most hot day in East Asia is strongly influenced by the anomalous anticyclonic circulation accompanying thermodynamic processes during the boreal summer^21–24^. Anomalous anticyclonic circulation leads to decreased cloud cover with an increase of downward solar radiation, resulting in the occurrence of hot day^25–27^. Meanwhile, the anomalous anticyclonic circulations in East Asia are caused by an eastward-propagating stationary wave train such as the circumglobal teleconnection, the Artic-Siberian plain teleconnection pattern and Scandinavia teleconnection pattern^28,29^. They may also be caused by a northward-propagating stationary wave train such as the Pacific-Japan pattern^24,30^. Indeed, sea surface temperature (SST) variability in the eastern tropical Pacific and Indian Oceans, which is related to the activity of Pacific-Japan pattern, strongly influences East Asian climate, including the surface temperature^31–35^. For example, anomalously warm conditions occur over the East and Southeast Asian landmass during El Niño. In particular, positive temperature anomalies occur over most of the East Asia in spring during the decaying period of El Niño^34^. Recent studies further argued that wave-train hot day, which occur simultaneously in Europe and East Asia, are increasing over time^36^. Indeed, the correlations of hot day occurrence and the Scandinavia pattern have increased over the past few decades^28^.

While there have been a number of studies of East Asia’s hot day in summer^18,24–30,36^, there are few studies on the occurrence of hot day in the boreal spring (March–April–May). In fact, there are some reports of a sharp increase in hot day occurrence during spring^37,38^. For instance, in 2014, South Korea experienced the hottest spring in 61 years and daily maximum temperatures exceeded 40 °C in many parts of northern China in 2014^37,38^. East Asia also experienced an unusual hot spring in 2018, when unprecedented surface temperatures were recorded across most of the East Asian region^39,40^.

These phenomena require further research to examine the physical processes on the characteristics of East Asia’s hot day in spring. In fact, there are previous studies which argue that mid-latitude stationary wave trains can be induced by SST anomalies in the North Atlantic and extend from the Atlantic to Eurasia^41–44^ through the atmospheric teleconnection pattern. The purpose of this study is to investigate the role of North Atlantic sea surface temperature (SST) on the increase in the East Asia’s hot day occurrence frequency in spring from 1979 to 2021.

## Results

### East Asia’s hot day occurrence frequency and North Atlantic Tripole (NAT)-like SST

**Figure 1:**
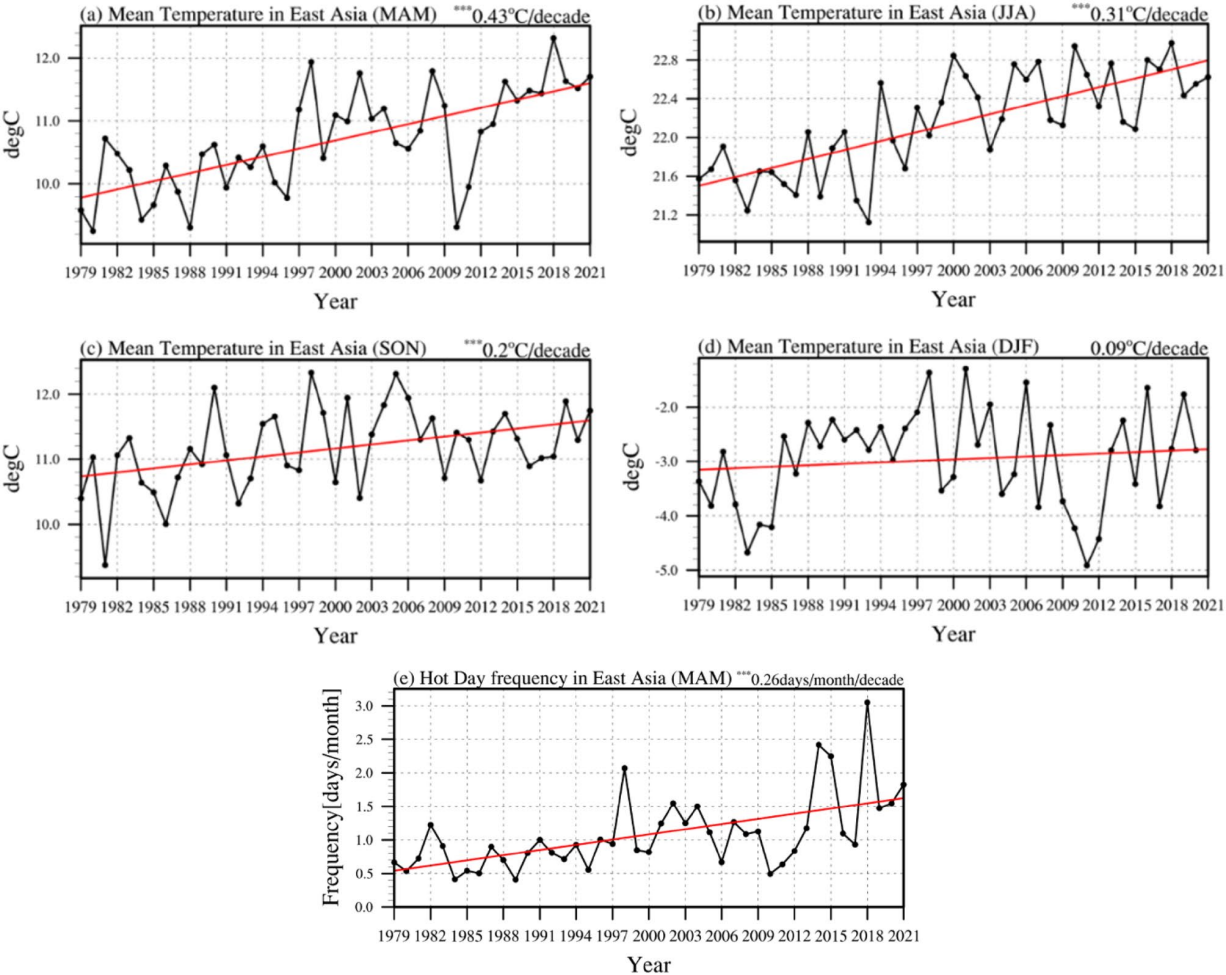
The time series of surface mean temperature and East Asia’s hot day frequency in boreal spring. The time series of surface mean temperature (°C) in East Asia for 1979–2021: (**a**) MAM (March–April–May), (**b**) JJA (June–July–August), (**c**) SON (September–October-November), and (**d**) 1979–2020 DJF (December–January–February). (**e**) Monthly mean of hot day frequency in boreal spring for 1979–2021. The red line denotes a linear trend and the value on the upper right corner is the linear trend’s regression coefficient. The 99% statistical significance level is marked by ***.

We plotted the time series of the surface temperature averaged in East Asia (20° N–50° N, 100° E–145° E) in each of the four seasons (Fig. 1a–d). The most notable feature is that the surface temperature in spring (Fig. 1a) showed a linear trend with a significant increase of 0.43 °C per decade, which is the largest trend among the four seasons. To examine the details, we calculated the spatial distribution of the surface temperature and hot day occurrence frequency (see Methods) in the Northern Hemisphere during the boreal spring (Supplementary Fig. 1a,b). Consistent with the results in Fig. 1a and e, the spatial structure of surface temperature is characterized by a significant warming trend in most regions of the Northern Hemisphere including East Asia (Supplementary Fig. 1a). Concurrently, the hot day occurrence frequency also shows an increasing trend in most regions including East Asia. (Supplementary Fig. 1b). This implies that the warming of land surface temperature is closely associated with the increase of the hot day occurrence frequency in spring, which is largely consistent with the notion that the increase of surface mean temperature accompanies the increase of the hot day occurrence^25–27,45^. Unless stated otherwise, hereafter, the results in the present study are mainly focused on the boreal spring.

Figure 1e displays the time series of hot day occurrence frequency in East Asia for 1979–2021. Indeed, there is a statistically significant increase of hot day occurrence frequency with the 99% confidence level, i.e., 0.26 days month^−1^ per decade, during the entire analyzed period. Note that a simultaneous correlation coefficient between the East Asian surface temperature (Fig. 1a) and hot day occurrence frequency (Fig. 1e) is 0.76 and 0.61 with and without a liner trend, respectively, which is statistically significant at the 99% confidence level. In particular, East Asia’s hot day occurrence frequency significantly increases after the early 2000s compared to before the early 2000s (Fig. 1e). The mean hot day occurrence frequency is 0.82 days per month for 1979–1999, and 1.33 days per month for 2000–2021, respectively, and their difference is statistically significant at the 95% confidence level. We argue that there is a significant increase of hot day occurrence frequency in East Asia across the 2000s.

There are a few studies which emphasized the role of North Atlantic SST forcing on East Asian climate via atmospheric teleconnections^46,47^. For example, there was an unprecedented warming observed in East Asia at 2018 during boreal spring, which was linked to the North Atlantic tripole (NAT)-like SST forcings^40^. The NAT-like SST forcings triggered anomalous Rossby wave trains propagating into East Asia across the Eurasia continent, resulting in anticyclonic circulation over East Asia along with significant warming. Based on these findings, we also examined how the hot day occurrence frequency in East Asia is related to the North Atlantic SST variability.

**Figure 2:**
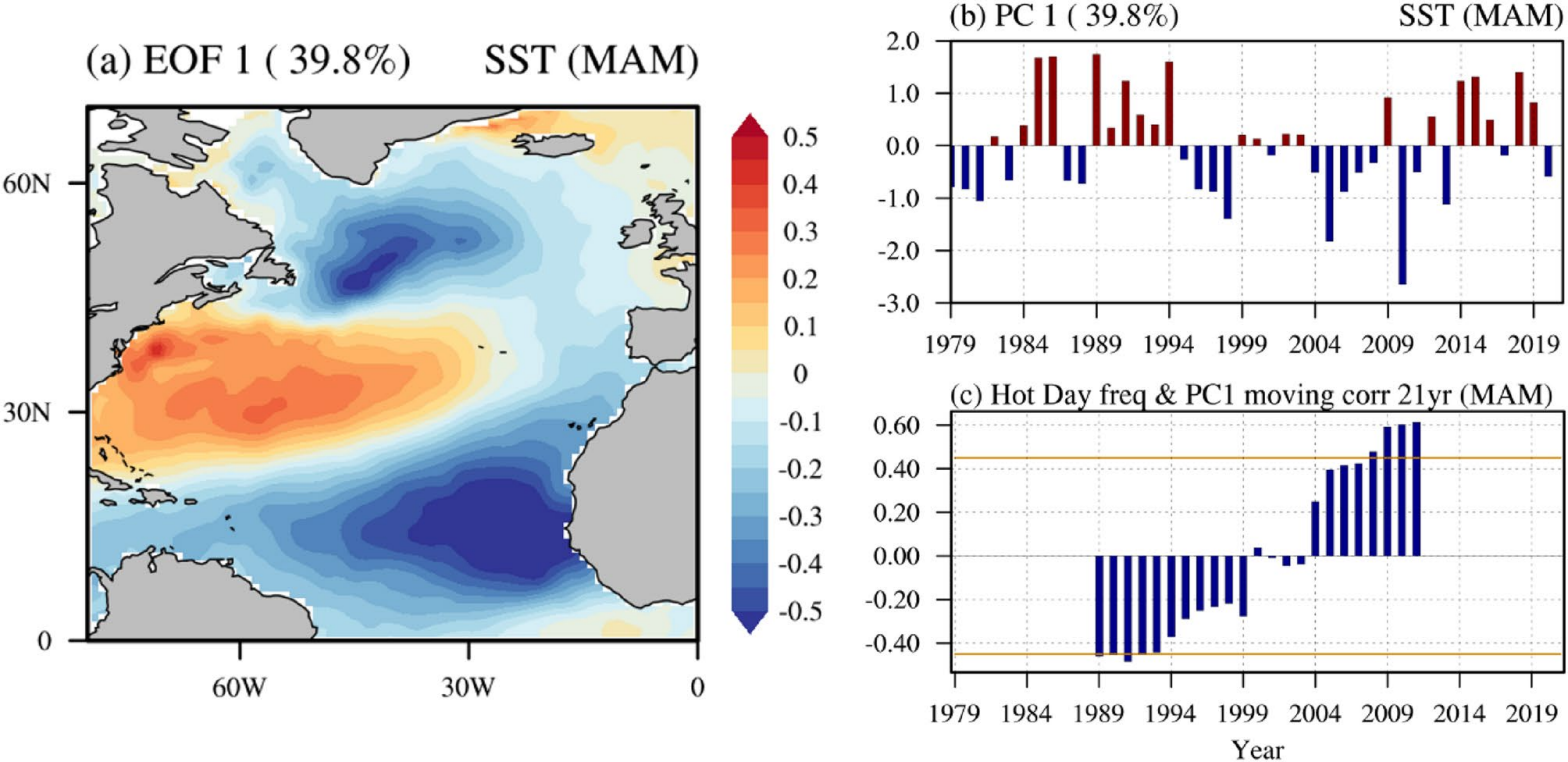
A positive phase of the NAT SST mode and the relationship with East Asia’s hot day frequency. (**a**) The first EOF of SST anomalies (°C) in the Atlantic Ocean (0°–75° N, 0°–80° W) for 1979–2021 in spring (**b**) The standardized principal component time series corresponding to the first EOF. The top-left corner is the fraction of explained variance to the total variance. (**c**) The blue bars show a 21-year moving correlation of principal component and East Asia’s hot day variability. The yellow line denotes the statistical significance at the 95% level.

To extract the dominant mode of SST variability in the North Atlantic Ocean (0°–75° N, 0°–80° W) in spring, we obtained the first empirical orthogonal function (EOF) mode using the SST anomalies without a linear trend (Fig. 2a). The first EOF SST mode is characterized by SST anomalies of alternating negative, positive, and negative from low latitude to high latitude in the North Atlantic Ocean, which is referred to as a positive phase of the NAT SST mode, hereafter. Figure 2b displays the principal component time series of the NAT SST mode for 1979–2021, which is characterized by the variability on low frequency timescales. Figure 2c displays the 21-year moving correlations between the NAT SST mode’s principal component time series (Fig. 2b) and the East Asia’s hot day occurrence frequency (Fig. 1e) from 1979 to 2021. The most striking feature is that the relationship of the NAT SST mode and East Asia’s hot day occurrence frequency is not stationary, but changes as time progresses (Fig. 2c). Prior to the early 2000s, East Asia’s hot day occurrence frequency tends to decrease during a positive phase of the NAT SST mode, however, it is not statistically significant in most periods. In contrast, the increase in the hot day occurrence frequency is accompanied with a positive phase of NAT SST mode after the early 2000s. This positive relationship of the East Asia’s hot day occurrence frequency and a positive phase of the NAT SST mode has strengthened in the recent past (Fig. 2c).

It is noteworthy that the variability of NAT SST mode is similar to that of the North Atlantic Oscillation (NAO) (Methods). Indeed, the NAO index and NAT SST mode is statistically significantly correlated with a correlation coefficient value of 0.55 for 1979–2021 (Supplementary Fig. 2a). We further show the 21-year moving correlation between the two indices, indicating that that their significant relationship is nearly stationary during the entire analyzed period (Supplementary Fig. 2b). Nevertheless, we used the NAT SST mode instead of the NAO index because the NAO index is not statistically significantly correlated with the East Asia’s hot day frequency in spring, i.e., a simultaneous correlation coefficient is 0.12 for 1979–2021.

### The role of the NAT SST mode across the early 2000s

To understand the role of NAT SST mode on the hot day occurrence in East Asia since the early 2000s, we divided the entire analyzed period into P1 (1979–1999) and P2 (2000–2021). The correlation coefficient between the East Asia’s hot day frequency and NAT-like SST principal component time series is -0.46 during P1 and 0.60 during P2. Indeed, the two periods have an opposite relationship, and the correlation coefficient is statistically significant at the 99% significance level during P2 only, implying that the North Atlantic SST plays a role in the occurrence of hot days in East Asia during P2.

**Figure 3:**
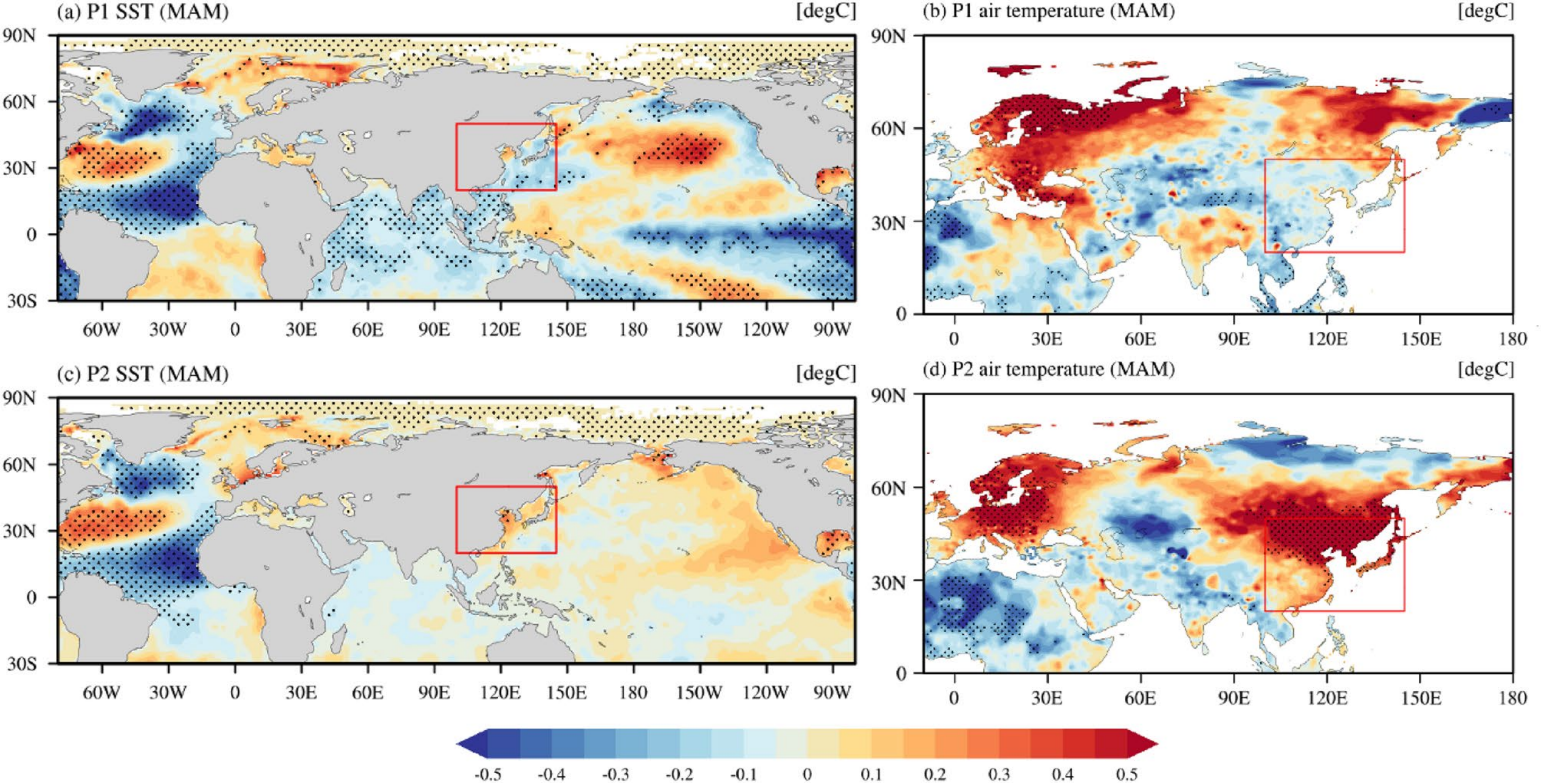
The regressed SST anomalies and surface air temperature anomalies against NAT SST mode. The regressed sea surface temperature anomalies (°C) against the principal component time series for (**a**) P1 (1979–1999) and (**c**) P2 (2000–2021). The black dots indicate 95% confidence level areas. The anomalies were calculated by subtracting (**a**) P1 and (**c**) P2 climatology data. The red box represents the region we investigated, East Asia (20° N–50° N, 100 °E–140° E). (**b**, **d**) Same as (**a**, **c**), except for the regressed surface air temperature.

We plotted the regressed SST anomalies against the NAT SST mode’s principal component time series during P1 and P2, respectively (Fig. 3a and c). It is evident that the regressed North Atlantic SST anomalies during both P1 and P2 periods (Fig. 3a and c) are quite similar to the spatial structure of a positive phase of the NAT SST mode, as shown in Fig. 2a. However, there are some distinct features outside the North Atlantic Ocean. A positive phase of the NAT SST mode during P1 is strongly associated with the anomalous cool SST in the Indian Ocean and La Niña-like SST anomalies in the eastern tropical Pacific (Fig. 3a). However, there are no statistically significant regressed SST anomalies associated with the NAT SST mode outside the North Atlantic during P2 (Fig. 3c). This implies that the NAT SST mode solely influences East Asia’s hot day occurrence during P2 compared to that during P1. Indeed, a positive phase of the NAT SST mode is associated with a cool surface temperature in East Asia during P1 although the statistical significance is negligible (Fig. 3b). In contrast, it is closely associated with the anomalous warm surface temperature in East Asia during P2 (Fig. 3d). The results in Fig. 3b and d are consistent with the results in Fig. 2c, which shows that a positive phase of the NAT SST mode is associated with the increase of hot day occurrence frequency as well as the surface warming in East Asia during P2 (Fig. 3d), in contrast to that during P1 (Fig. 3b).

**Figure 4:**
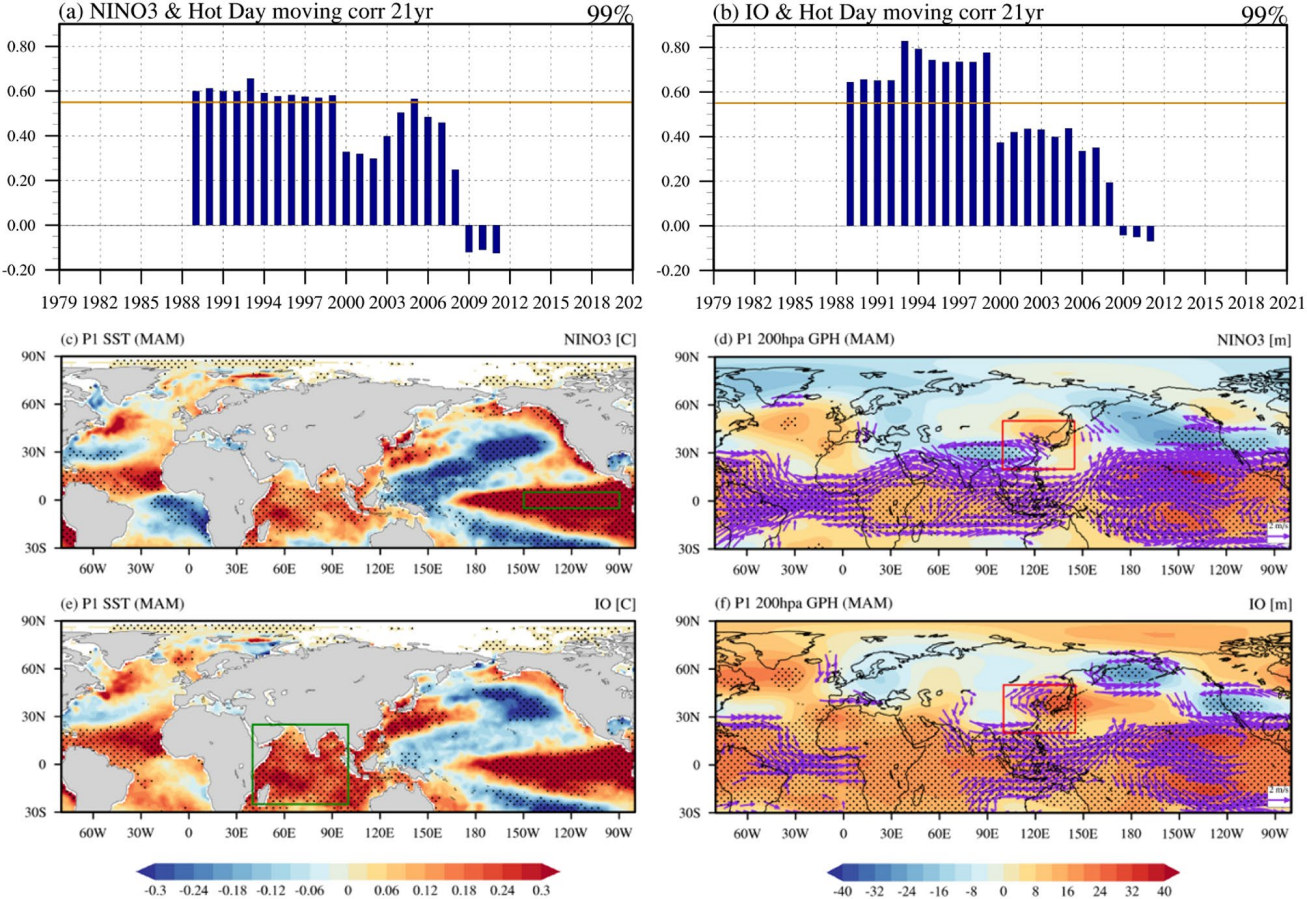
The relationship of East Asia’s hot day frequency and the eastern tropical Pacific and Indian Ocean. Same as Fig. 2c, except for (**a**) NINO3 (5° S–5° N, 90°–150° W) and (**b**) Indian Ocean (25° S–25° N, 40°–100° E). (**c**) Map of regressed SST anomalies (°C) against NINO3 SST anomalies time series without the linear trend for period P1 (1979–1999). The black dots indicate 95% confidence level areas. (**d**) Same as (**c**), except for geopotential height (m) and wind (m s^−1^). The winds significant at the 95% confidence level are marked with a purple vector. The anomalies were calculated by subtracting P1 climatology data. The green box and red box in (**c**, **d**) represent the NINO3 (5° S–5° N, 90°–150° W) region and East Asia (20° N–50° N, 100° E–140° E), respectively. (**e**, **f**) Same as (**c**, **d**), except for Indian Ocean (25° S–25° N, 40°–100° E).

The SST variability in the eastern tropical Pacific and Indian Ocean largely influences East Asian climate including the surface temperature^31–35^. Therefore, in addition to the NAT SST mode, we calculated the 21-year moving correlation coefficient between the East Asia’s hot day occurrence frequency and the SST anomalies in the NINO3 region (5° S–5° N, 90° W–150° W) (Fig. 4a) and the Indian Ocean region (25° S–25° N, 40° E–100° E) (Fig. 4b) without the linear trend. It is found that the anomalous warm SST in the eastern tropical Pacific SST anomalies as well as the Indian Ocean are associated with the increase of East Asia’s hot day occurrence frequency before the early 2000s, but no significant correlation is seen after the early 2000s. In addition, the anomalous warm SST in the eastern tropical Pacific (Fig. 4c) and the Indian Ocean (Fig. 4e) during P1 are associated with a negative phase of the NAT SST mode, which is consistent with the result in Fig. 3a. Concurrently, the anomalous warm SST in both the eastern tropical Pacific and Indian Ocean are associated with the anticyclonic circulation that favored anomalous warm surface temperatures over East Asia and vice versa (Fig. 4d and f). Investigating the relationship of the NAT SST mode and the eastern tropical Pacific and the Indian Ocean SST is beyond the scope of the current study. However, the results in Fig. 4 may indicate that a negative relationship of a positive phase of the NAT SST mode and East Asia’s hot day occurrence frequency (Fig. 2c) as well as East Asian surface temperature (Fig. 3b) is largely influenced by the role of the eastern tropical Pacific and the Indian Ocean SST on East Asia’s hot day occurrence frequency during P1.

**Figure 5:**
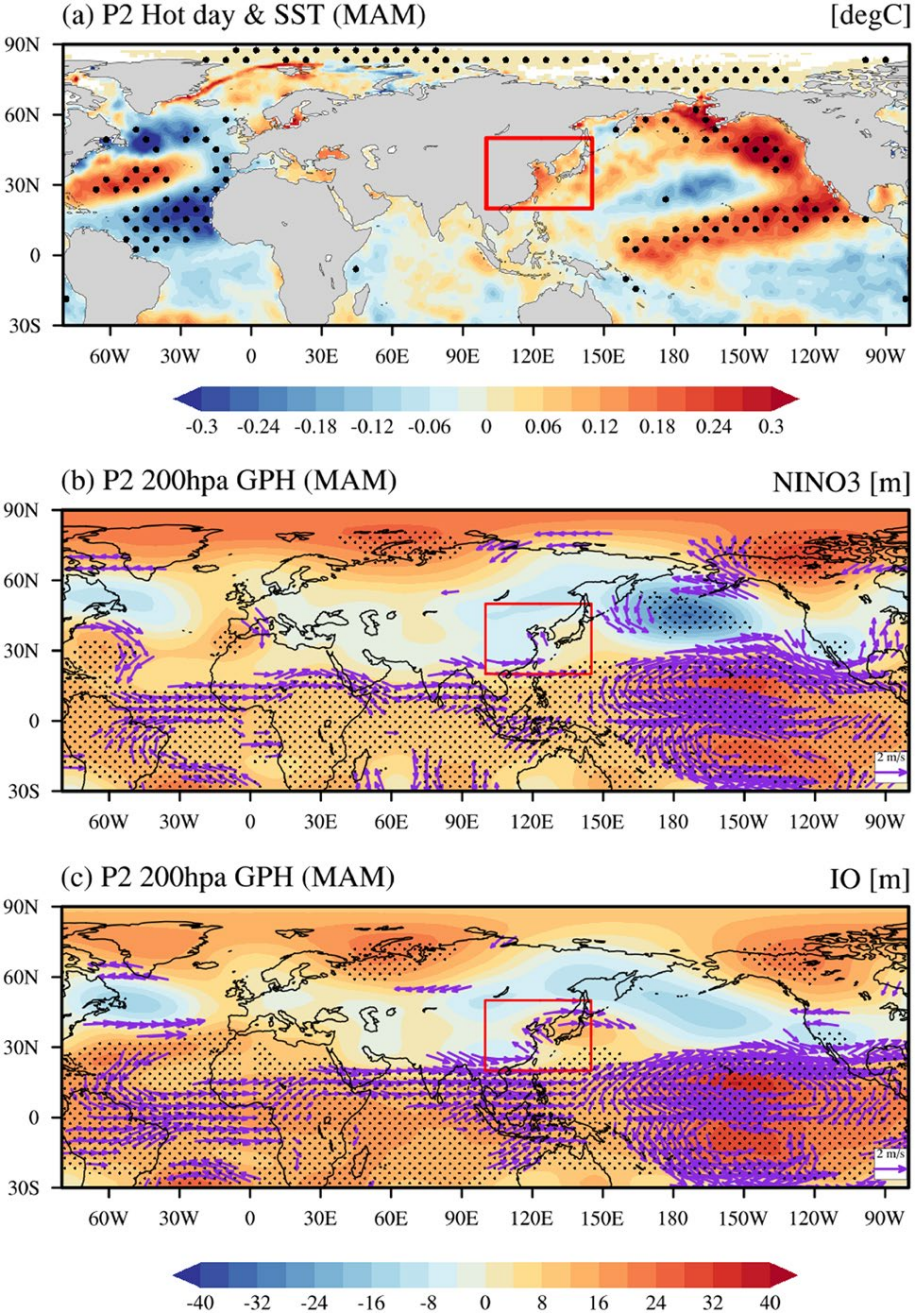
The regressed SST, GPH and horizontal winds anomalies. (**a**) The regressed SST anomalies (°C) against the East Asian hot day variability for P2 (2000–2021). (**b**), (**c**) is the regressed geopotential height (meter) and horizontal winds (m s^−1^) anomalies at 200 hPa against with the NINO3 SST index and Indian Ocean SST for P2 (2000–2021). The black dots indicate 95% confidence level areas.

On the other hand, the SST forcing, which could be associated with the East Asian hot day occurrence, is observed in the central-to-eastern subtropical Pacific with a tilted structure after 2000s (Fig. 5a). Figures 5b,c show the regressed geopotential height anomalies at 200 hPa against with the SST anomalies averaged in the NINO3 (5° S–5° N, 90°–150° W) and Indian Ocean (25° S–25° N, 40°–100° E), respectively. In fact, the anomalous anticyclonic circulation associated with the NINO3 SST and the Indian Ocean SST is not statistically significant in East Asia. This result is consistent with the result in Fig. 5a. We speculate that the atmospheric teleconnections, which is associated with the SST forcing over the Indian Ocean and the eastern equatorial Pacific, have been changed during the 2000s. This results in their weakening relationship with the occurrence of East Asian hot days after the 2000s.

We additionally analyzed large-scale atmospheric circulation based on the regressed geopotential height and horizontal wave activity flux (Methods) at the 200 hPa level against the NAT SST mode’s principal component time series (Supplementary Fig. 3a and b). The most notable difference between the two periods is that the horizontal stationary wave train from the North Atlantic to East Asia is significant in the upper atmosphere during P2 only (Supplementary Fig. 3b) but not during P1 (Supplementary Fig. 3a). During P2, the anomalous upper atmosphere anticyclonic circulation originating from the North Atlantic Ocean into East Asia is observed (Supplementary Fig. 3b). This implies that the atmospheric stationary waves forced by a positive phase of the NAT SST forcing propagate into East Asia, leading to the anticyclonic circulation over East Asia during P2. This results in the hot day occurrence as well as the anomalous warm surface temperature during P2 (see Fig. 3d). In fact, there are some previous studies which argue that mid-latitude stationary wave trains can be induced by SST anomalies in the North Atlantic and extend from the Atlantic to Eurasia^41–44^, and in particular, the SST-induced heating over the North Atlantic Ocean is capable of generating the atmospheric teleconnection pattern similar to the East Atlantic/West Russian teleconnection^43^.

We further found that the spatial distribution of the North Atlantic SST linear trend is very similar to a positive phase of the NAT SST mode (Supplementary Fig. 4 and Fig. 2a). This suggests that the linear trend of North Atlantic SST, which might be due to internal variability or global warming or their combination, may play a role to strengthen the impact of a positive phase of the NAT SST mode on the East Asia’s hot day occurrence during P2, as shown in Fig. 2c.

### Linear Baroclinic model experiment

We hypothesize that the NAT SST forcing may induce the zonally oriented horizontal stationary wave train into East Asia, leading to an increase of hot day occurrence during P2. To support this notion, we perform an idealized Linear Baroclinic Model (LBM) experiment^48^ (Methods). Note that we used the climatological (1979–2021) atmospheric variables to obtain a basic state to conduct an idealized LBM experiment. The experiment was aimed to examine the role of heating rate between the two periods (P2 minus P1) under the climatological (1979–2021) state of the atmospheric variables.

**Figure 6:**
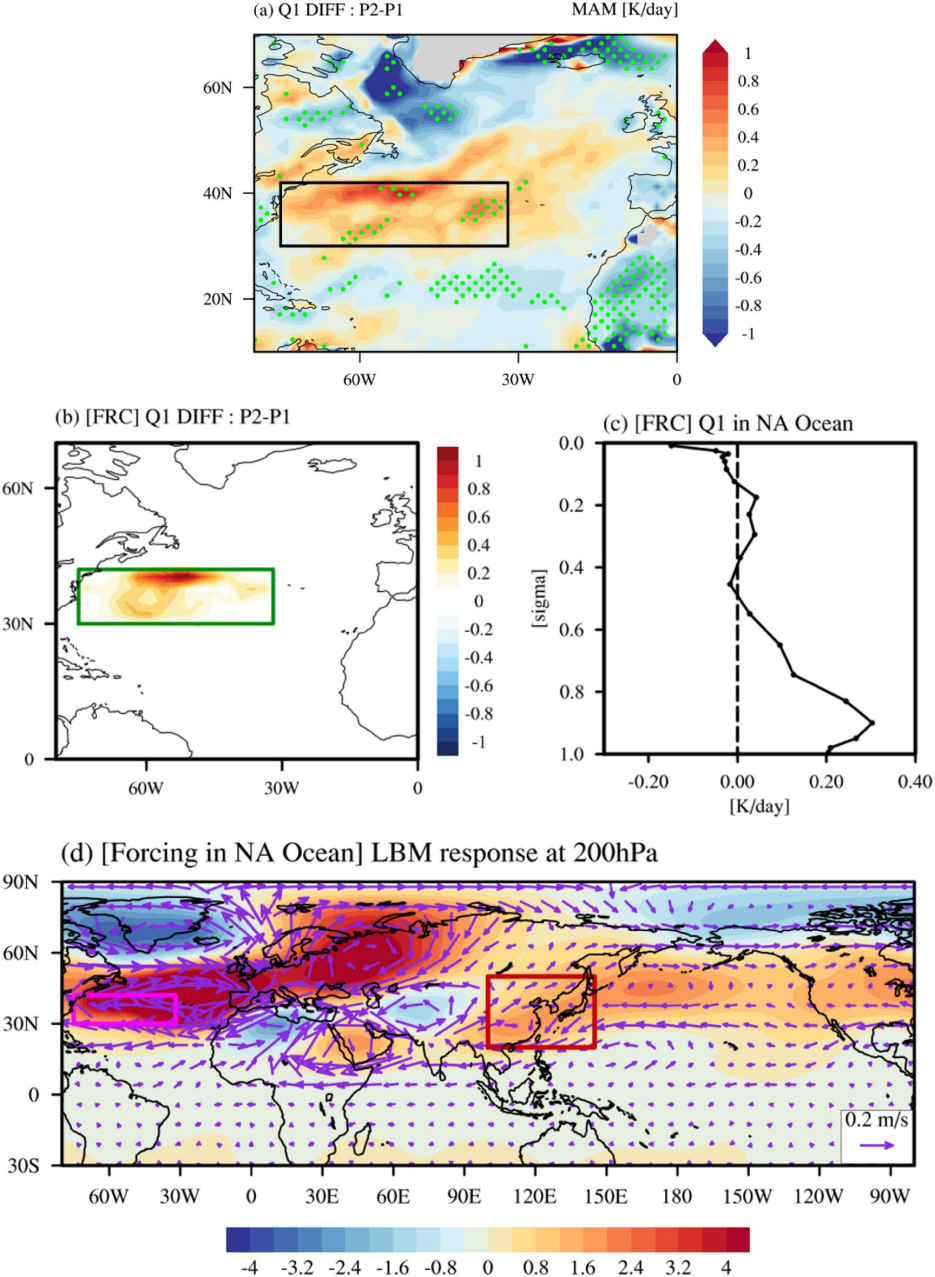
Results of the LBM experiment. (**a**) Difference of heating rates averaged vertically from 1000 hPa to 850 hPa for each period of P1 and P2 when PC1 in Fig. 2b is greater than a 0.5 standard deviation. The black box represents the region we investigated (30° N–42° N, 75° W–32° W). The areas significant at the 90% confidence level are marked with green dots. (**b**) The thermal forcing (K day^−1^) prescribed to the LBM experiment with the same results as those obtained from the reanalysis data. The green box in (**b**) represents the region we investigated (30° N–42° N, 75° W–32° W), which is same as black box in (**a**). (**c**) The vertical profile of the forcing in the green box. (**d**) The result of the LBM experiment obtained as an average of 20 days after reaching steady state for 60 days. The color shading represents geopotential height (meter) and the purple vectors show wind speed (m s^−1^) and direction. The magenta box and red box represent the region we investigated (30° N–42° N, 75° W–32° W) and East Asia (20° N–50° N, 100° E–140° E), respectively.

Figure 6a displays the difference in heating rates averaged vertically from 1000 hPa to 850 hPa between P1 and P2 in which the NAT SST mode’s principal component time series are greater than a 0.5 standard deviation. We found that the spatial distribution of this difference is similar to the NAT SST mode and we selected the western North Atlantic Ocean (30° N–42° N, 75° W–32° W) where the heating rate is higher during P2 than that during P1 to force the LBM. A higher heating rate during P2 than P1 could be related to the liner trend of North Atlantic SST in which the spatial pattern is similar to that of a positive phase of the NAT SST mode (Supplementary Fig. 4 and Fig. 2a). Note that the heating rate difference without a linear trend between P2 and P1 (Supplementary Fig. 5) is quite similar to that with a linear trend (Fig. 6a) although the difference is not statistically significant.

The difference of heating rates obtained from the reanalysis data over selected regions (Fig. 6a) was used to conduct the LBM experiment (Fig. 6b,c). It was found that the composite map of geopotential height anomalies and wind anomalies, which was obtained from the LBM experiment, are characterized by anomalous anticyclonic circulation over East Asia (Fig. 6d). This result is comparable to that from the reanalysis data (Supplementary Fig. 3b), supporting that a positive phase of the NAT SST mode during P2 is effective to induce the eastward propagating Rossby wave train, contributing to the development of anomalous anticyclone circulation in East Asia. This results in the increase of hot day occurrence frequency in East Asia during P2. It is noteworthy that the similar results were obtained from the previous studies^41,42^ in which Atmospheric General Circulation Models are used to examine the role of North Atlantic SST, indicating that the present study may not be dependent of model complexity.

## Summary and discussion

In summary, we found that the correlation between NAT SST mode and East Asia’s hot day frequency changed from negative to positive across the early 2000s and the East Asia’s hot day frequency is statistically significantly related to a positive phase of the NAT SST mode during P2 only. We argued that the stationary wave train associated with a positive phase of the NAT-like SST forcing is effective to induce an anomalous anticyclonic circulation over East Asia favorable to hot day occurrence during P2. To support this notion, we conducted the LBM experiment in which the difference in heating rates over the North Atlantic Ocean between the two periods was prescribed to force the LBM and obtained a similar result compared to the reanalysis dataset. This result indicates that the eastward propagating stationary wave train originating from the North Atlantic Ocean during P2 induces anomalous anticyclonic circulation in the upper atmosphere over East Asia, which led to an increase in East Asia’s hot day occurrence. Furthermore, the spatial distribution of the North Atlantic SST linear trend is similar to that of a positive phase of the NAT SST mode. We speculate that the higher diabatic heating rate during P2 than that during P1 is associated with the North Atlantic SST trend.

On the other hand, there is a statistically significant positive correlation between the East Asia’s hot day occurrence frequency and anomalous warm SST including the eastern tropical Pacific and Indian Ocean during P1 only. We argue that the atmospheric teleconnections, which is associated with the SST forcing over the Indian Ocean and the eastern equatorial Pacific, have been changed during the 2000s. This results in their weakening relationship with the occurrence of East Asian hot days during P2.

Finally, we found that East Asian hot day frequency shows no statistically significant trend for period 1948–1978 (Supplementary Fig. 6a) and the relationship between the East Asia’s hot day occurrence frequency and the NAT SST mode is nearly stationary (Supplementary Fig. 6b–d). A simultaneous correlation coefficient between them is -0.41 during the entire analyzed period (1948–1978), which is statistically significant at the 95% confidence level. This result also supports the notion that the relationship between the NAT SST mode and the occurrence of hot days in East Asia has changed after the 2000s.

## Methods

### Observational and reanalysis datasets

The records of daily temperature were obtained from the National Oceanic and Atmospheric Administration Climate Prediction Center (NOAA CPC) dataset. The dataset’s maximum temperature (T_max_) and minimum temperature (T_min_) have a horizontal resolution of 0.5° × 0.5°. The monthly SST dataset was obtained from the Hadley Centre Sea Ice and Sea Surface Temperature with a horizontal resolution of 1.0° × 1.0° for 1979–2021^49^. The reanalysis datasets were obtained from the National Centers for Environmental Prediction—Department of Energy Reanalysis 2^50^ with a horizontal resolution of a 2.5° × 2.5° and 17 pressure levels for the period of 1979–2021 and the National Centers for Environmental Prediction/National Center for Atmospheric Research (NCEP/NCAR) reanalysis data 1^51^ with a horizontal resolution of a 2.5° × 2.5° and 17 pressure levels for the period of 1948–1978. We also used the Japanese 55-year Reanalysis with 1.25° resolution and 37 pressure levels^52^ to obtain atmospheric variables for heating rates. The North Atlantic Oscillation (NAO) index is obtained from obtained from NOAA.

### Definition of Hot day occurrence

The thresholds to define hot day were calculated based on the 90^th^ percentile of daily T_max_ and T_min_ (1991–2020) at each grid point. Hot day is defined as day when both T_max_ and T_min_ are equal to or exceed the threshold (Supplementary Table 1). We calculated the monthly hot day mean frequency at each grid from 1979 to 2021 during the boreal spring. We averaged the hot day frequency in East Asia (20° N–50° N, 100° E–145° E) according to the IPCC report ^53^.

### Wave activity flux

In order to examine the distribution of stationary Rossby wave train propagation, we used the wave activity flux (WAF) developed by Takaya and Nakamura^54,55^. The horizontal WAF is defined as follows:

1 $$W = \frac{p\cos \phi }{{2\left| U \right|}}\left[ {\begin{array}{*{20}c} {\frac{U}{{a^{2} \cos^{2} \phi }}\left\{ {\left( {\frac{{\partial \psi^{\prime}}}{\partial \lambda }} \right)^{2} - \psi^{\prime}\frac{{\partial^{2} \psi^{\prime}}}{{\partial \lambda^{2} }}} \right\}} \\ { + \frac{V}{{a^{2} \cos \phi }}\left\{ {\frac{{\partial \psi^{\prime}}}{\partial \lambda }\frac{{\partial \psi^{\prime}}}{\partial \phi } - \psi^{\prime}\frac{{\partial^{2} \psi^{\prime}}}{\partial \lambda \partial \phi }} \right\}} \\ {\frac{U}{{a^{2} \cos \phi }}\left\{ {\frac{{\partial \psi^{\prime}}}{\partial \lambda }\frac{{\partial \psi^{\prime}}}{\partial \phi } - \psi^{\prime}\frac{{\partial^{2} \psi^{\prime}}}{\partial \lambda \partial \phi }} \right\}} \\ { + \frac{V}{{a^{2} }}\left\{ {\left( {\frac{{\partial \psi^{\prime}}}{\partial \lambda }} \right)^{2} - \psi^{\prime}\frac{{\partial^{2} \psi^{\prime}}}{{\partial \phi^{2} }}} \right\}} \\ \end{array} } \right]$$

The variables $a\left( { = 6.37 \times 10^{6} m} \right)$, $\phi$, and $\lambda$ are the Earth’s radius, latitude, and longitude, respectively. The variables p, U, and V denote the climatology (1991–2020, March–April–May) of geopotential, zonal wind, and meridional wind at the 200 hPa level, respectively. The geostrophic stream function perturbation is defined as $\psi^{\prime} = \frac{g}{f}\phi^{\prime}$, where $\phi^{\prime}$ is the regressed geopotential height anomalies during boreal spring against the principal component time series of NAT SST mode, $g\left( { = 9.81\;{\text{m}}^{2} {\text{s}}^{ - 1} } \right)$ is the gravitational acceleration, and $f\left( { = 2{\Omega }\sin \phi } \right)$ is the Coriolis parameter with the Earth’s rotation rate ${\Omega }\left( { = 7.29 \times 10^{ - 5} rads^{ - 1} } \right)$. The details of WAF can be found at https://github.com/laishenggx/T-N_Wave-Activity-Flux.

### Linear baroclinic model

In order to examine the effect of diabatic heating on the steady atmospheric response, we used the linear baroclinic model (LBM)^48^. The LBM is based on dry dynamical core and primitive equations linearized about a basic state on a sphere with a horizontal resolution of the T42 Gaussian grid and 20 vertical levels of sigma coordinates. The T42 is a regular longitude/latitude global horizontal grid with approximately 2.8° resolution. The results of the model experiment are presented as an average of 20 days after reaching steady state for 60 days. The climatological means of background fields were derived from NCEP/DOE Reanalysis 2 data for 1979–2021. The diabatic heating prescribed in LBM is represented by two radiative heating rates (shortwave heating rate and longwave heating rate) and three non-radiative heating rates (large scale condensation heating rate, convective heating rate, and eddy vertical diffusion heating rate) obtained from JRA-55^56,57^.

### Supplementary Information

Supplementary Information.

## Data Availability

All the datasets are freely available on public domain; National Oceanic and Atmospheric Administration Climate Prediction Center (https://psl.noaa.gov/data/gridded/data.cpc.globaltemp.html). Hadley Centre Sea Ice and Sea Surface Temperature (https://www.metoffice.gov.uk/hadobs/hadisst/). National Centers for Environmental Prediction—Department of Energy Reanalysis 2. (https://psl.noaa.gov/data/gridded/data.ncep.reanalysis2.html). National Centers for Environmental Prediction/National Center for Atmospheric Research (NCEP/NCAR) reanalysis data 1 (https://psl.noaa.gov/data/gridded/data.ncep.reanalysis.html). Japanese 55-year Reanalysis. (https://rda.ucar.edu/datasets/ds628.1/). NAO index (https://www.ncei.noaa.gov/access/monitoring/nao/).

## Code availability

The codes used in this study are available upon request to the first author Yong-Han Lee (yhlee27@hanyang.ac.kr). All the figures in the main text and supplementary information are generated by NCAR Command Language (NCL) version 6.4.0. NCL can be found at public domain (https://www.ncl.ucar.edu/). The LBM code is available from http://ccsr.aori.u-tokyo.ac.jp/~lbm/sub/lbm.html.
