# Supplementary material for: The role of the North Atlantic Ocean on the increase in East Asia’s spring extreme hot day occurrences across the early 2000s
Source: Sci Rep. 2024 Apr 30;14:9872. doi: 10.1038/s41598-024-59812-y (PMC11058825; doi:10.1038/s41598-024-59812-y)
Supplement: Supplementary file 1 — Supplementary Information. [file 41598_2024_59812_MOESM1_ESM.docx]

**Supplementary Information**

**The role of the North Atlantic Ocean on the increase in East Asia’s spring extreme hot day occurrences across the early 2000s**

Yong-Han Lee^1^, Sang-Wook Yeh^1*^, Jeong-Hun Kim^2^ and Maeng-Ki Kim^2^

^1^Department of Marine Science and Convergent Technology, Hanyang University, Ansan 15588, South Korea

^2^Department of Atmospheric Sciences, Kongju National University, Gongju, 32588, South Korea

^*^*Corresponding author: Prof. Sang-Wook Yeh, (E-mail:* [*swyeh@hanyang.ac.kr*](mailto:swyeh@hanyang.ac.kr)*), Hanyang University, Ansan, South Korea*

**This file includes:**

Supplementary Table 1

Supplementary Figures 1-5

**Introduction**

This supporting information provides the figures as mentioned in the main article.

**Supplementary Table 1. Extreme hot day (hot day) definition.**

|  | hot day |
| --- | --- |
| Definition | $T_{\max}$ ≥ 90^th^ percentile threshold of climatological $T_{\max}$  and  $T_{\min}$ ≥ 90^th^ percentile threshold of climatological $T_{\min}$ |

**
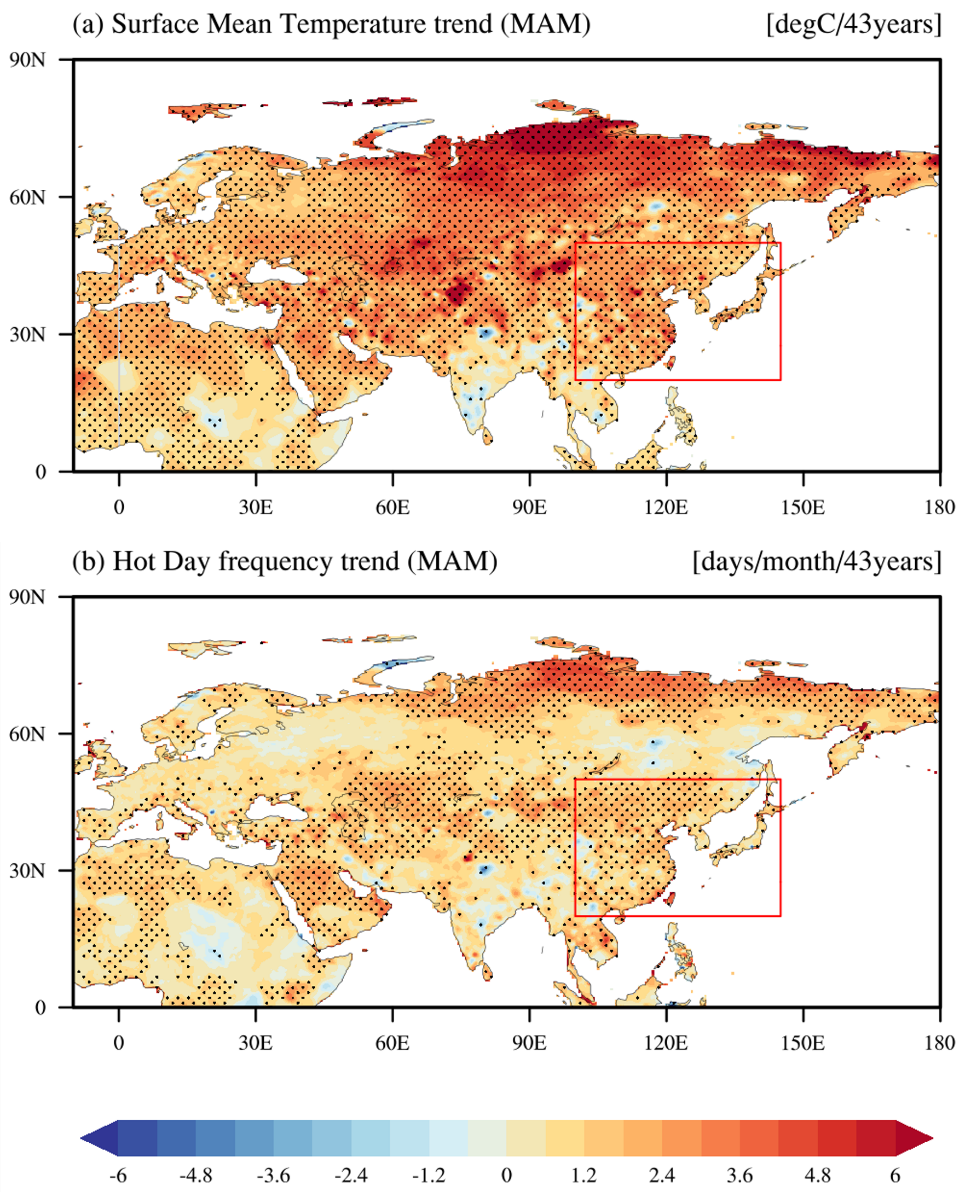
**

**Supplementary Figure 1. The linear trend of surface mean temperature and hot day occurrence frequency in Northern Hemisphere during the boreal spring (March-April-May).** (a) The linear trend of the surface mean temperature (Unit: ℃ per 43 years) and (b) hot day occurrence frequency (Unit: days month^-1^ per 43 years) in boreal spring for 1979-2021. Red box in (a)-(b) denotes East Asian region (20$^{\circ}$N-50$^{\circ}$N, 100$^{\circ}$E-140$^{\circ}$E). Black dots in (a)-(b) indicate the statistical significance at the 95% confidence level.

**
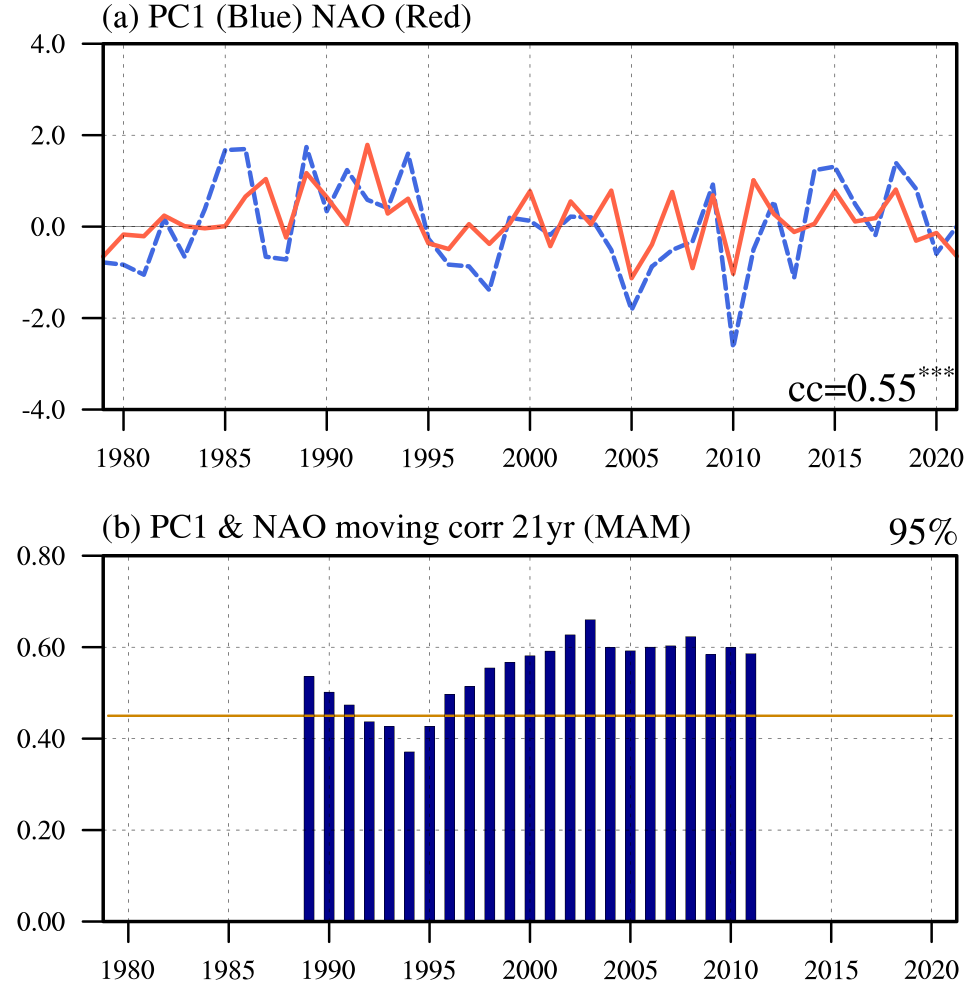
**

**Supplementary Figure 2. The NAT SST mode and the NAO index** (a) NAO index (red solid line) and NAT SST mode principal component (blue dashed line). “cc” in the under right corner is the two indices’ correlation coefficient. The 99% statistical significance level is marked by ***. (b) A 21-year moving correlation of NAT SST mode principal component time series and the NAO index. The yellow solid line denotes the statistical significance at the 95% level.

**
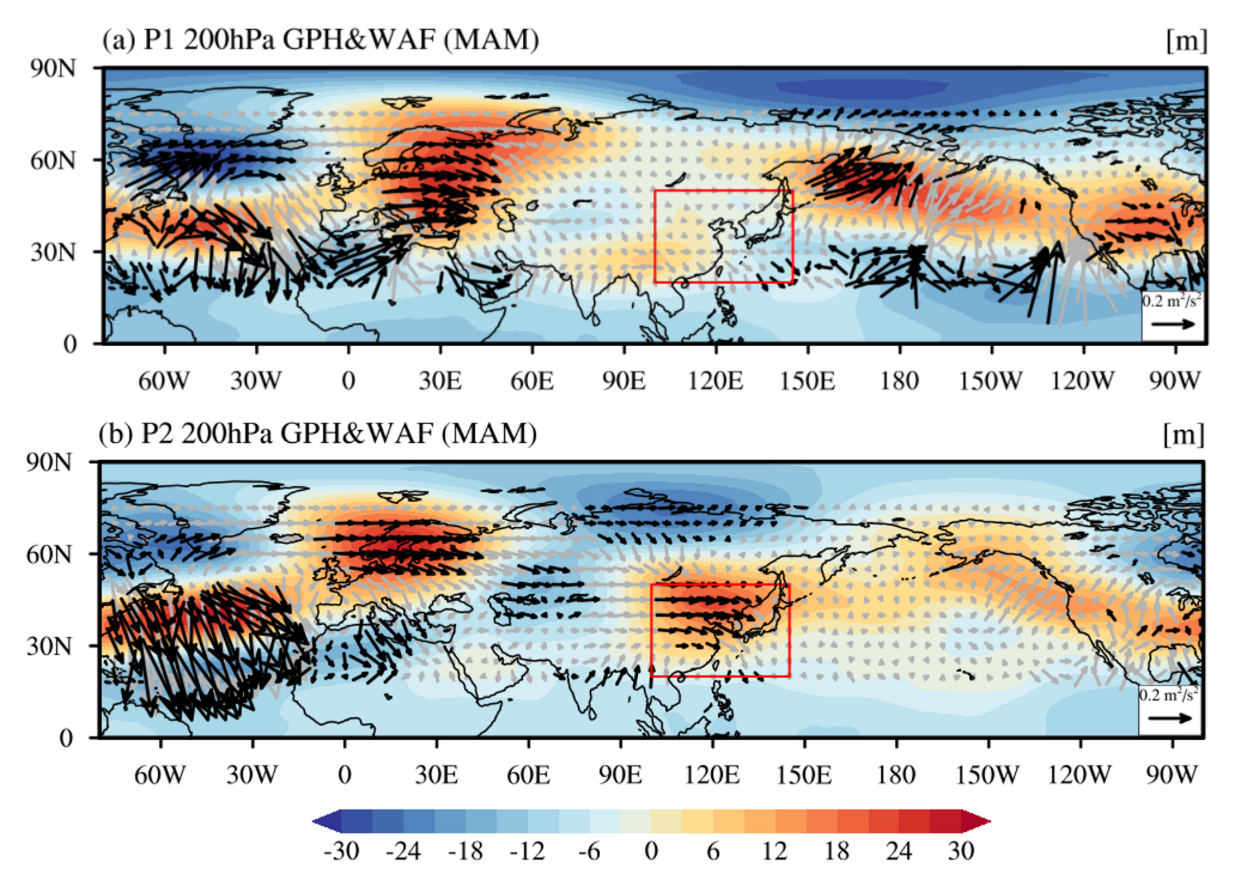
**

**Supplementary Figure 3. The regressed wave activity flux and geopotential height at 200hPa against NAT-like SST pattern.** The regressed geopotential height anomalies (color shading, m) and wave activity flux (black vectors, m^2^ s^-2^) at the 200 hPa level against with the principal component time series for (a) P1 (1979-1999) and (b) P2 (2000-2021). The areas significant at the 95% confidence level are marked with a black vector.


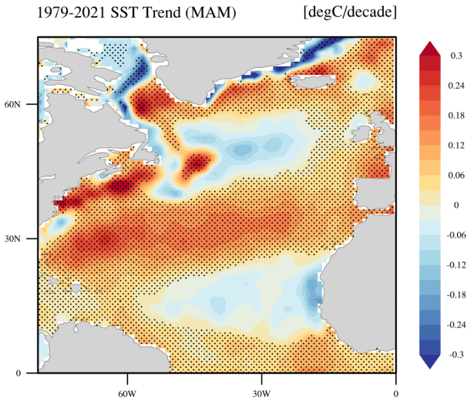


**Supplementary Figure 4. The linear trend of the North Atlantic SST in boreal spring.** The linear trend of the North Atlantic SST (℃ per decade) in spring for 1979-2021. The black dots indicate 95% confidence level areas. This was calculated by subtracting the area averaged global linear trend.

*
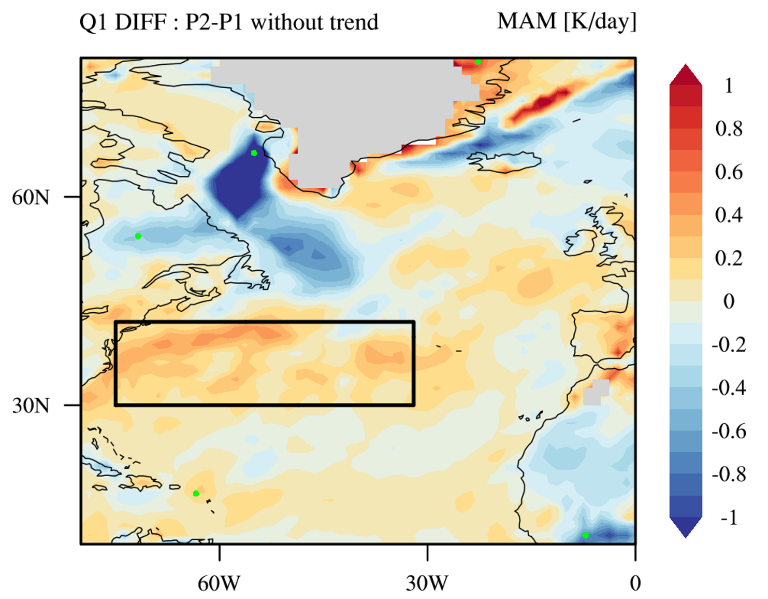
*

**Supplementary Figure 5. The difference in the heating rate between the period P1 and P2 without a linear trend.** Supplementary figure 5 is the same as in Fig. 6a in the main text except but without a linear trend.


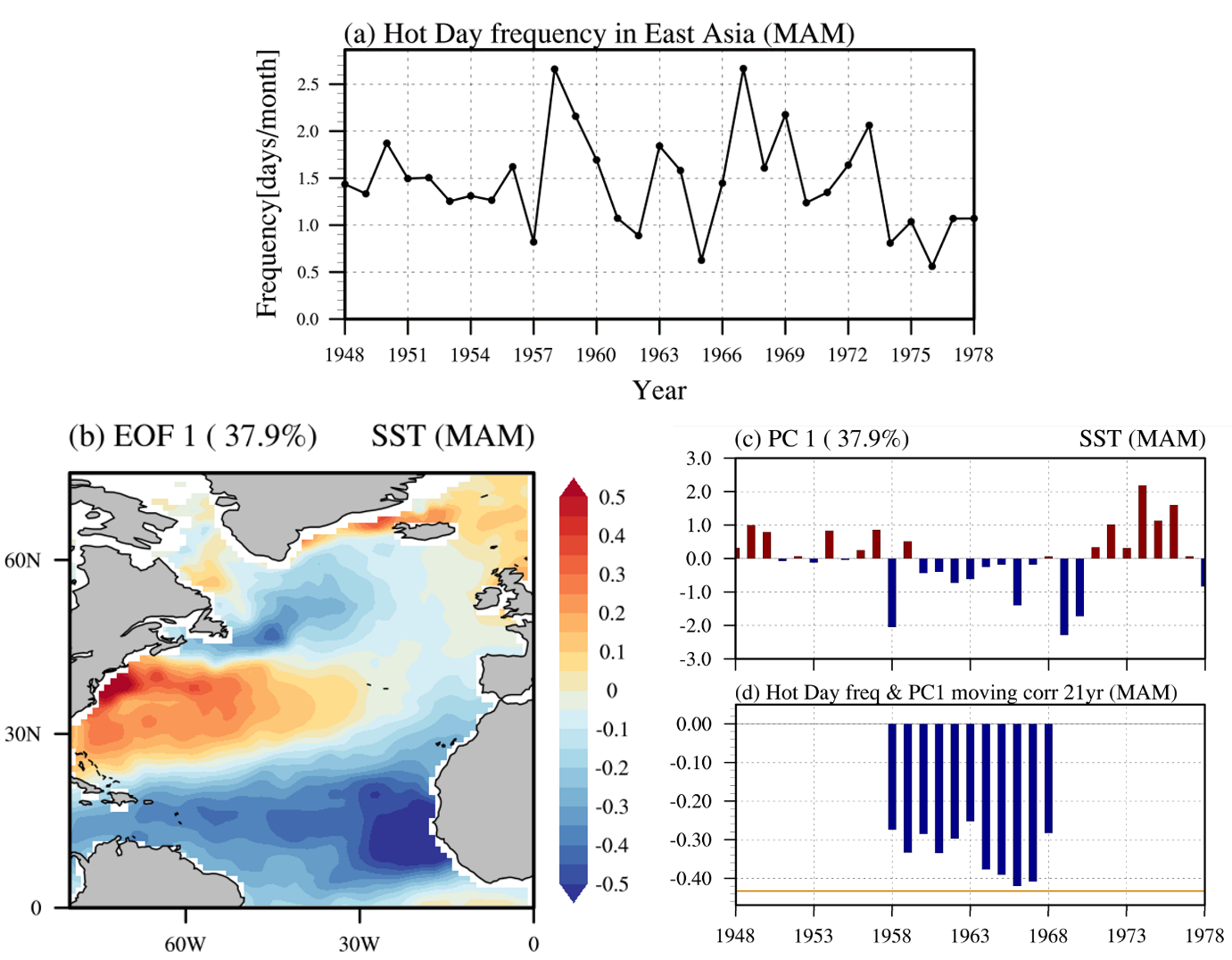


**Supplementary Figure 6. The East Asian hot day occurrence frequency obtained from reanalysis data.** (a) Monthly mean of hot day frequency in boreal spring for 1948-1978. (b) The first EOF of SST anomalies (℃) in the Atlantic Ocean (0°­-75°N, 0­°-80°W) for 1948-1978 in spring (c) The standardized principal component time series corresponding to the first EOF. The top-left corner is the fraction of explained variance to the total variance. (d) The blue bars show a 21-year moving correlation of principal component and East Asia’s hot day variability. The yellow line denotes the statistical significance at the 95% level.
